# Testing the Reliability and Validity of the Social Isolation Visual Analogue Scale Among Older Adults

**DOI:** 10.1093/geroni/igaf122.2790

**Published:** 2025-12-31

**Authors:** Anne Hagan, Barbara Resnick, Nicole Brandt, Sarah Holmes, Jennifer Klinedinst

**Affiliations:** UMB SON, ELKRIDGE, Maryland, United States; University of Maryland, Baltimore, Maryland, United States; UMB School of Pharmacy, 20 N. Pine St. Baltimore, Maryland, United States; University of Maryland School of Nursing, Baltimore, Maryland, United States; UMB SON, 655 W. Lombard Street, Baltimore, Maryland, United States

## Abstract

**Background and Purpose:**

The purpose of this study was to test the reliability and validity of the Social Isolation Visual Analogue Scale.

**Methods:**

This was a descriptive study. Forty residents from four senior housing communities were interviewed related to social isolation, depression, and social networks at baseline and two weeks later.

**Results:**

The mean age of the participants was 78.8 (SD = 8.1), the majority were female (86%) and Black (60%). There was evidence of test-retest reliability with a significant correlation between baseline and two-week follow-up of the Social Isolation Visual Analogue Scale (r=.74, p<.05). There was a significant relationship between the Social Isolation Visual Analogue Scale and depression and social networks.

**Conclusions:**

The findings from this study provided some preliminary evidence of the reliability and validity of the Social Isolation Visual Analogue Scale in low-income senior housing residents. Keywords; Social isolation, older adults, senior housing, social networks

